# Prevalence and predictors of diabetes-related distress in adults with type 1 diabetes

**DOI:** 10.1038/s41598-022-19961-4

**Published:** 2022-09-21

**Authors:** Natasa Grulovic, Martina Rojnic Kuzman, Maja Baretic

**Affiliations:** 1 Sanofi, Paris, France; 2 Department of Psychiatry and Psychological Medicine, University Hospital Centre Zagreb, School of Medicine, University of Zagreb, Zagreb, Croatia; 3 Department of Endocrinology and Diabetes, Internal Clinic, University Hospital Centre Zagreb, School of Medicine, University of Zagreb, Zagreb, Croatia

**Keywords:** Psychology, Diseases, Endocrinology, Health care

## Abstract

Type 1 diabetes (T1DM) is a chronic disease requiring lifelong insulin therapy and rigorous self-management. As it negatively impacts the affected individuals’ quality of life, it may eventually lead to diabetes-related distress. This study evaluated the prevalence and identified the predictors of diabetes-related distress in a representative sample of adults with T1DM treated at secondary and tertiary levels in Croatia. A multicenter, cross-sectional study was conducted in adults with T1DM in Croatia (N = 100). Data were collected between January 2018 and December 2018 from medical records and interviews during a single clinical visit, when participants completed a 20-item Problem Area in Diabetes (PAID) Questionnaire. The proportion of participants with a total PAID score ≥ 40 indicating high diabetes-related distress was calculated, and binary logistic regression was run to determine predictors. High diabetes-related distress was found in 36% of participants, with a mean PAID total score of 31.9 (21.1). The predictors of diabetes-related distress were higher HbA1c level (OR = 1.491, p = 0.037, CI = 1.025–2.169) and the presence of microvascular complications (OR = 4.611, p = 0.005; 95%CI 1.546–13.754). Worrying about the future and chronic complications and feeling guilty when off-track with diabetes management were identified as items that contribute the most to distress. Diabetes-related distress is a frequent condition in adults with T1DM in Croatia. Special attention should be given to patients with suboptimal glycemic control and microvascular complications. Given the high prevalence and impact of psychosocial problems in diabetes, psychological care should be integrated into routine care for adults with type 1 diabetes.

## Introduction

Type 1 diabetes mellitus (T1DM) is a chronic condition caused by autoimmune destruction of insulin-secreting pancreatic β-cells, characterized by severe insulin deficiency. T1DM impacts a patient’s physical health status and increases their overall psychosocial burden. There are many complex environmental, social, behavioral, and emotional factors that influence living with diabetes. For adults living with T1DM, a chronic disease that requires constant self-management, emotional problems are a common occurrence^1^.

Diabetes-related distress reflects the person’s emotional response to the burden of living with a largely self-managed chronic disease and its complications^2^. Diabetes-related distress in people with type 2 diabetes is a prominent condition with an overall prevalence of 36%, associated with female gender and comorbid depression^3^ and a poorer quality of life^4^. Some studies confirmed that elevated diabetes-related distress was experienced by 20–30% of people with T1DM, suggesting a widespread clinical problem in this population as well^5^. Unlike type 2, the onset of T1DM is linked to a younger age and is often associated with stressful life events; psychosocial factors were shown to play a role in both its etiopathogenesis and disease management^6^. Diabetes-related distress in adults with T1DM is associated with suboptimal glycemic control and tends to be higher for women and relatively younger adults^2^. Prolonged, significant distress in chronic disease like T1DM is further associated with an increased prevalence of depressive symptoms^7^.

T1DM requires lifelong insulin therapy and constant strict self-management. The presence of diabetes-related distress in patients with T1DM might present barriers to adequate self-management and overall treatment outcomes^8^. Minimizing psychosocial burden and consequently, diabetes-related distress is one of the goals of type 1 management^9^. Specific diagnostic tools for screening diabetes-related distress are being developed. One of them is Problem Areas in Diabetes questionnaire (PAID)^10^, recently recommended within a standardized set of validated psychosocial measures by the International Consortium for Health Outcomes Measurement (ICHOM)^11^. PAID scale covers a great variety of emotional concerns, has been validated in research and clinical settings, and is available in 17 languages^12^. While T1-DDS is a version of the DDS specifically designed for people with type 1 diabetes, its availability is currently limited to English, French, German, Portuguese, and Spanish translated and validated versions^13^. In our study participants were asked to self-complete a paper questionnaire during this visit, translated and linguistically validated Croatian version of PAID was used.

The overall number of people with diabetes registered in the Croatian National Diabetes Registry in 2020 was 310,212^14^. The approximate population with type 1 diabetes is 20,000. Croatia is a European country with publicly funded healthcare, meaning it is accessible to everyone. For the T1DM treatment with multiple daily injections, both second generation insulin analogs are available. The use of diabetes technology is increasing as continuous glucose monitoring (CGM) is available without a copayment for people living with T1DM. Usage of insulin pumps is still relatively low (roughly 10% of patients having type 1 diabetes) but is rising along with the closed loop usage. Individual studies on the prevalence and, more often, on predictors of diabetes-related distress solely in the adult T1 population are scarce and often limited by small sample sizes^15^. Additionally, to our best knowledge, there are no reports in the literature about the burden of diabetes-related distress in adults with T1DM in Croatia. This study aims to evaluate the prevalence of such distress in adults with T1DM distress in a representative sample of adults with T1DM treated at secondary and tertiary levels in Croatia, and to identify the predictors of diabetes-related distress in this population.

## Subjects and methods

This study is nested within the ‘SAGE’ study, a larger multinational, cross-sectional, observational study of glycemic control, hypoglycemia, and diabetes management in T1DM conducted in 17 countries across Asia, Eastern Europe, Latin America, the Middle East, and Western Europe^16^.

The study was carried out at hospital centers in Croatia, between January 2018 and December 2018. To secure a representative sample, investigators were selected randomly (computer-generated randomization) from all Croatian endocrinologists and diabetologists employed at secondary and tertiary institutions in Croatia where adults with T1DM are being treated. The potential investigators and participating physicians were contacted by phone in ascending order from the randomized list and selected for the study if they agreed to participate. The process continued until a target number of five eligible physicians was reached. The reasons for non-participation were: physician were not reachable by phone in three attempts, physicians rejected to participate due to disinterest, and physicians were engaged with other studies with the same population. These rejections were recorded in the call log. Once the physician agreed to participate, he/she recruited the first 20 eligible patients consecutively within a two-month period. In total, 100 participants were invited to participate in the study. 0 of them refused to participate, 0 did not meet the eligibility criteria. Included participants fulfilled the eligibility criteria defined by study protocol: they were diagnosed with T1DM for ≥ 1 year, aged ≥ 26 years, with recent HbA1c available within the 30 days preceding the study visit. Exclusion criteria were diabetes other than T1DM, change in insulin therapy within three months preceding the study, and non-insulin treatment at any time since T1DM diagnosis. All participant provided written informed consent. A screening log form was completed by the physician to document the site’s selection process of the study patients. Participating hospitals were General Hospital Pula, General Hospital Varazdin, Vuk Vrhovac University Clinic for Diabetes, Endocrinology and Metabolic Diseases, University Hospital Merkur, and Clinical Hospital Center Rijeka. The Agency for Medicinal Products and Medical Devices of Croatia’s Central Ethics Committee’s approval was obtained. All experiments were performed in accordance with relevant guidelines and regulations.

During the single visit at the time of the study, data (age, gender, level of education, single-person or non-single-person household, duration of the T1DM, presence of chronic complications, A1C value, hypoglycemia occurrence) were collected from patient’s records and interviews. The physicians, using the patients’ medical records, completed a case report form (CRF) with yes/no questions about the patients’ microvascular complications, including the presence of neuropathy, retinopathy, or nephropathy. The number of documented symptomatic hypoglycemic episodes (blood glucose ≤ 3.9 mmol/L and blood glucose ≤ 3.0 mmol/L) during the last three months was recorded in CRF. After data collection, participants were asked to self-complete the Problem Areas in Diabetes (PAID) questionnaire, which were afterwards collected by the project staff.

### Questionnaire

**Figure 1:**
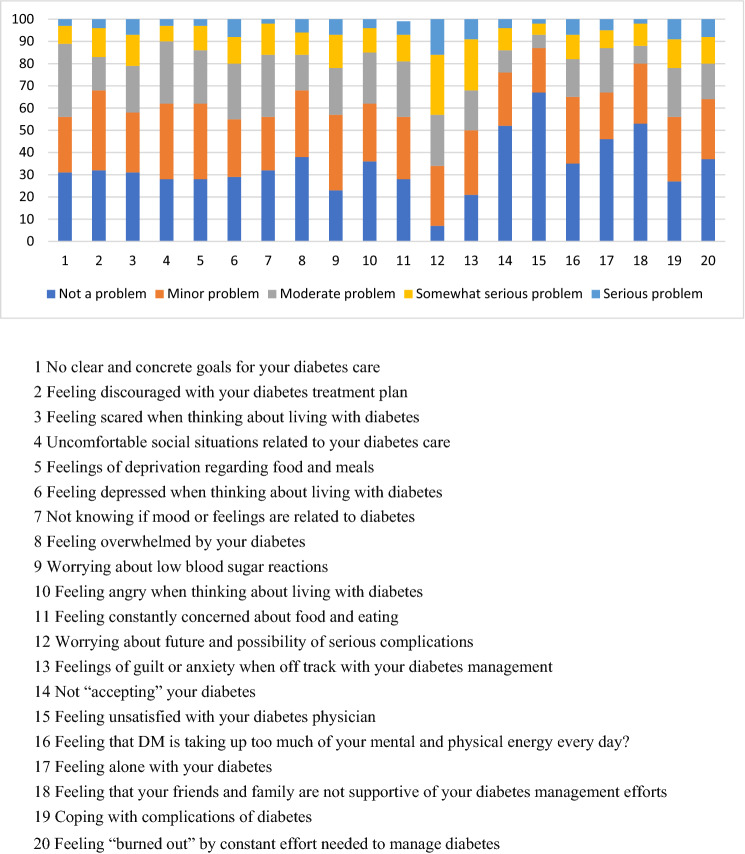
Rates of responses to individual PAID Items.

For this study, we used the Problem Areas in Diabetes (PAID) questionnaire, more specifically, its Croatian version of February 6, 2018^17^. PAID is a 20-item screening instrument designed to measure emotional responsiveness specific to diabetes^10^. The items, as listed in Fig. 1, are rated on a 5-point Likert scale and respondents indicate the degree to which each of the items is currently a problem for them; 0 (not a problem), 1 (minor problem), 2 (moderate problem), 3 (somewhat serious problem), 4 (a serious problem). The scores for each item are summed, then multiplied by 1.25 to generate a total score out of 100. Higher total score indicates higher distress. A total PAID score ≥ 40 was considered as high diabetes-related distress^18^.

### Statistical methods

Data was analyzed using statistical software SPSS (IBM, V 25.0)^19^. The normality of distribution was verified using the Shapiro–Wilk test.

Descriptive analysis was applied to establish the patients’ characteristics and to define the proportion of patients with T1DM with a PAID score ≥ 40. Binary logistic regression analysis was conducted with PAID total score categorized above cut-off or below, and with following variables as possible predictors: age, gender, duration of T1DM diabetes, presence of microvascular complications, hypoglycemia occurrence, HbA1c. Regression was done with the simultaneous entry of all predictors to examine the effect of each predictor when adjusting for variance shared between all predictors. All variables were entered at the same time with criterion 0.05 for variable entry and 0.10 for removal.

## Results

100 patients fulfilled the eligibility criteria and were included in the analysis. Participants' characteristics are shown in Table 1. The mean PAID total score in our study sample was 31.92 (21.14) with a high degree of internal consistency (Cronbach alpha = 0.95). High diabetes-related distress defined as a PAID total score ≥ 40 was found in 36% of participants.

**Table 1:** Participants’ characteristics.

Participants’ characteristics (N = 100)	Mean (SD) or %	Range
Current age (years)	48.11 (15.53)	26.0–81.0
Gender (% female)	48%	
**Duration of T1DM (years) **	20.92 (13.15)	1.09–54.00
< 10 years (n, %)	25 (25%)	
≥ 0 years (n, %)	75 (75%)	
HbA1c (%)	7.29 (1.28)	4.20–12.70
**Insulin treatment**		
Multiple daily injections (%)	86	
Pump use (%)	14	
PAID total score	31.92 (21.14)	0.0–83.75
PAID total score ≥ 40, proportion of patients	36%	
**Education**		
Primary/secondary	55.7%	
University/higher education	44.3%	
**Living conditions**		
Alone	6%	
With another adult	94%	

Predictors for PAID scores greater than 40 are shown in Table 2. Patients with presence of microvascular complications have 4.6 times higher risk for PAID score above 40 (OR = 4.611, p = 0.005; 95%CI 1.546–13.754). HbA1c increase for 1% increases risk for higher PAID score for 1.5 times (OR = 1.491, p = 0.037, CI = 1.025–2.169). Other predefined sociodemographic and diabetes variables were not correlated with diabetes-related distress.

**Table 2:** Predictors of high diabetes-related distress (total PAID score ≥ 40).

	P	OR	95% CI
sex(M)	0.379	1.529	0.594–3.941
age.r	0.216		
age.r (< 40)	0.112	2.875	0.783–10.562
age.r (41–60)	0.770	1.196	0.361–3.956
duration_cat (< 10 years)	0.175	0.395	0.104–1.511
HbA1c	0.037	1.491	1.025–2.169
Hypoglycemia 3.9r (yes)*	0.411	0.568	0.147–2.188
Hypoglycemia 3.0r (yes)**	0.602	0.732	0.226–2.366
Microvascular (yes)	0.006	4.611	1.546–13.754
Constant	0.008	0.015	

*Patients with at least one symptomatic hypoglycemia with blood glucose ≤ 3.9 mmol/L within the last 3 months.

**Patients with at least one symptomatic hypoglycemia with blood glucose ≤ 3.0 mmol/L within the last 3 months.

Responses rates to individual PAID items are shown in Fig. 1. Of the 100 subjects, 59% reported serious concerns for at least one PAID item. Items which most of the participants perceived as distressing (i.e., items which scored 3 or 4) were item 12 “Worrying about the future and complications” (43% of participants) and item 13 “Feeling guilty when off-track with management” (32% of participants). In addition, several items were highly scored by 20% or more participants. Those were item 3 “Feeling scared when thinking about living with diabetes”, item 6 “Feeling depressed when thinking about living with diabetes”, item 9 “Worrying about low blood sugar reactions”, item 19 “Coping with complications”, item 20 “Feeling burnt-out by the constant effort needed to manage diabetes”. Items scored by most participants with 0 (“not a problem”) are as follows: item 4 “Not “accepting” your diabetes” (52% of participants), item 15 “Feeling unsatisfied with your diabetes physician” (67%), item 17 “Feeling alone with your diabetes” (46%) and item 18 “Feeling that your friends and family are not supportive of your diabetes management efforts” (53%).

## Discussion

We found that more than one-third of our study sample suffered from substantial diabetes-related distress. Previous studies showed that elevated diabetes-related distress affects 20–30% of people with T1DM, with the range difference recorded in prevalence across different populations and healthcare systems from 8 to 65%^5^. Our results are concordant with the study in the USA which reported prevalence of diabetes-related distress in T1DM of 42.1%^20^. The same study showed that, among those with elevated diabetes-related distress at baseline, 71% report similarly high levels at nine month follow-up. Interestingly, we found that the duration of the disease did not predict diabetes-related distress. Several explanations are possible. For example, the source of distress could have changed over time, as in the example where duration is strongly associated with both complications and hypoglycemia risk. Alternatively, it may indicate that adaptation to distress in persons with T1DM is not a matter of time, as a passive process, but that it requires the person to actively cope with the illness and accept the changes in life that are associated with the occurrence of DM. For example, to accept their own fears of the complications instead of denying it and not adhering to the diet, new healthy lifestyle etc. This may indirectly indicate that a psychosocial intervention may be needed to help the person cope with diabetes-related distress. This may be especially important for those with prolonged distress, as it can predispose to problematic self-care behavior^7^. Indeed, severe diabetes-related distress increases the chances of poor treatment outcomes and the risk of diabetes-related complications^21^. Of course, other factors such as general coping abilities and life circumstances (for example poor socioeconomic status) not assessed in this study that relate to diabetes distress may explain these results.

The mean PAID total score in our study was 31.92 (21.14) and is comparable to the results of SAGE study^22^.

The results of our study indicate that the presence of elevated HbA1c levels is a significant predictor of diabetes distress. This is concordant with the results of the T1 Exchange Clinic Registry in which HbA1c was one of the strongest predictors significantly associated with diabetes-related stress when adjusting for all other variables^15^.

It is possible that uncontrolled diabetes, defined by high HbA1c levels, elevates the distress in patients, as patients may be worried about the consequences of diabetes and the lack of success in the treatment, especially over a course of time. However, it is also possible that other features, such as anxiety or overwhelming distress in life, may confer to both the increase of stress related to diabetes and to elevated levels of HbA1c.

Concordant with our finding which indicates that the presence of elevated HbA1c levels is a significant predictor for diabetes distress, we also found that the presence of microvascular complications is also a significant predictor. First, we may assume that those with higher levels of HbA1c will also have a higher probability to develop microvascular complications^23^, indicating that (psychological) factors contributing to elevated HbA1c may result in contributing to microvascular complications over time. Secondly, it is also possible that acquiring microvascular complications lead to impairment of organ functioning that the patient feels through loss or impaired functioning or limitation in everyday life, and thus the fear of disease and potential impact on ability in the future as well distress increase. No other significant predictors for higher diabetes-related distress among sociodemographic and disease characteristics were found. While associations between diabetes-related distress and gender, decreased age, and diabetes duration were demonstrated elsewhere^15^, our study findings yield no difference in the level of diabetes-related distress among genders and age groups. A possible explanation could be the higher mean age of our study sample which was 48.11 (15.53) vs 37.64 (16.33) in T1 Exchange Clinic Registry. The second possibility is the different method of calculation, which in our study was binary logistic regression with the main variable being categorized as either above cut-off score or below, while the mentioned study used the original continuous PAID score variable. Interestingly, most of our study participants were worried about complications, (e. g., neuropathy, retinopathy, and nephropathy) and hypoglycemia, which are described as the most prevalent diabetes-specific fears in people with diabetes^24^, so intervention in patient education is justified.

In our study we found that some individual items in the PAID questionnaire were highly scored by majority of studied population, pointing to moderate or severe distress regarding a particular topic^25^. Worrying about the future and chronic complications and feeling guilty when off-track with diabetes management were the most prominent concerns, and these findings are comparable with the results of a previous study of diabetes-related distress made in Croatian population with both type 1 and type 2 diabetes participants^26^. Interestingly, feeling guilty when off-track with management was the most prominent description of feelings associated with distress, followed by feeling burnt-out by the constant effort needed to manage diabetes and feeling scared and depressed when thinking about living with diabetes, coping with complications and blood sugar levels, which may indicate the formation of the vicious cycle in which the patients with DM are caught in, by trying and failing to “control” their illness and future of it^27^. For example, their constant worrying about the complication of diabetes due to non-optimal glycemia levels and the negative predictions about the future of their illness increasing their level of fear/anxiety may result in the patients feeling burnt by the constant effort needed to manage diabetes (to control their illness – glycemia levels) – leading to increased depression and fear due to living with diabetes, which then increases the negative perceptions of the future forming the vicious cycle^28^. Alternatively, constant worrying about the complications and negative predictions about the future of their illness, fear and depression may also lead to denial of the potential effects of chronic diabetes mellitus, which results in them failing to adhere to diet/medication and leading to non-optimal glycemia and ultimately increasing the possibility of complications of DM, followed by feelings of guilt when off-track with diabetes management^29^. This will again increase their worrying about complications closing the vicious cycle. The way how diabetes-related distress manifests in the two different populations may be contextually different due to differences in age, predisposing conditions, treatment outcomes, and type of treatment. Our findings on commonly perceived distress items solely in T1DM population could be a signal to the clinicians on what to address in clinical consultation.

The importance of psychosocial care and a call for improved psychosocial outcomes are recognized by the American Diabetes Association which issued recommendations to integrate psychosocial care within patient-centered medical care, stressing that such care should be provided to all diabetic patients^30^. Furthermore, the recent Consensus Report on the management of T1DM acknowledged ongoing psychosocial support as a relevant component of T1DM management, as treatment outcomes are highly dependent on a person’s ongoing self-care behavior^9^. Notably, our findings suggest that social support availability is perceived as highly relevant by our study participants as more than 80% of participants reported scores < 3 to the associated item 18. Thus, psychosocial support could be a protective factor from diabetes related distress and perceived problems with self-management in adults with diabetes^31^. Screening and monitoring for psychosocial problems using patient-appropriate standardized and validated tools are recommended at the initial visit, and periodically thereafter if glycemic targets are not met and/or at the onset of diabetes complications. While the treatment of psychological aspects related to T1DM may be as important as the medical management in improving living with diabetes^32^, the method of delivering it is still unclear^33^.

The screening should be used to detect the overall levels of diabetes-related distress, at the very beginning of the treatment. Depending on the PAID scores, several interventions should be offered, in addition to the standard treatment, including education. For those with low to moderate levels of diabetes-related distress, education should be provided in an empathic form by the health care team treating diabetes, seeing as 67% of participants expressed satisfaction with their diabetes physician. For highly distressed adults with T1DM, having poor glycemic control, diabetes-related distress can be successfully addressed using both educational and emotion-focused approaches^34^. In addition, psychological or psychiatric liaison consultations should be available.

Considerable strengths of the study are the inclusion of a representative sample of T1DM patients treated at secondary and tertiary centers in Croatia and the usage of standardized, diabetes-specific measure that allows for replication of the study findings. Our results made solely in T1DM patients give greater clarity of understanding this condition in specific patients. Lastly, according to our knowledge, this is the first study of this kind in Croatia.

Limitations of this study include a cross-sectional design which implies interpretation and clinical recommendations should be made with caution. The sample size is likely too small to confirm the lack of association among many of the variables. Other comorbidities or life events that could influence distress levels were not assessed and evaluated in this study.

## Conclusion

The results of our study highlight the emotional burden of diabetes-related distress in people with T1DM in Croatia. Given the high percentage of persons with diabetes-related distress, it may suggest a serious, yet neglected clinical problem. It shows the vulnerability of this group of patients, who are worried the most about the future, developing complications and hypoglycemia. To overcome the identified problem, we recommend to; (1) Train the multidisciplinary team treating diabetes to deliver structural educational programs for all adults with T1DM about diabetes-related distress and about its influence on T1DM outcomes; (2) Screen for diabetes distress using the PAID standardized questionnaire; (3) Offer specific psychological interventions delivered by mental health professionals or liaising mental health professionals when needed. Addressing diabetes-specific emotional distress during clinical consultation and patient educational programs may empower individuals with T1DM to improve self-management of their disease thus contributing to treatment outcomes improvement.

## Data Availability

Raw data generated in the study are available in the open access repository Open Science Framework https://osf.io/ecpbu/.
